# A classical swine fever virus E2 fusion protein produced in plants elicits a neutralizing humoral immune response in mice and pigs

**DOI:** 10.1007/s10529-020-02892-3

**Published:** 2020-04-22

**Authors:** Youngmin Park, Sangmin Lee, Hyangju Kang, Minhee Park, Kyungmin Min, Nam Hyung Kim, Sungmin Gu, Jong Kook Kim, Dong-Jun An, SeEun Choe, Eun-Ju Sohn

**Affiliations:** 1 BioApplications Inc., Pohang Techno Park Complex, 394 Jigok-ro Nam-gu, Pohang, Korea; 2 Virus Disease Division, Animal and Plant Quarantine Agency, Gimcheon, 39660 Gyeongbuk Korea

**Keywords:** *Nicotiana benthamiana*, Classical swine fever virus, DIVA concept, Subunit vaccine, Molecular farming, Fc-fusion protein

## Abstract

Classical swine fever (CSF) is one of the most important viral diseases of swine worldwide. Although live or attenuated virus vaccines have been used to control CSFV, it is difficult to distinguish vaccinated pigs from infected pigs; this leads to restrictions on import and export. Subunit vaccines based on the CSFV E2 glycoprotein have been developed using baculovirus or insect cell systems, but some weaknesses remain. Here, we describe production of an E2 recombinant protein using a *Nicotiana benthamiana* plant expression system. To do this, we took advantage of the ability of the swine Fc domain to increase solubility and stability of the fusion protein and to strengthen immune responses in target animals. *N. benthamiana* expressed high amounts of pFc2-fused E2 proteins, which were isolated and purified by affinity chromatography to yield a high pure recombinant protein in a cost-effective manner. Native-polyacrylamide gel electrophoresis and size exclusion chromatography confirmed that the pmE2:pFc2 fusion exists as a multimer rather than as a dimer. Injection of recombinant pmE2 protein into mice or piglets generated anti-pmE2 antibodies with efficient neutralizing activity against CSFV. These results suggest that a purified recombinant E2 protein produced in *N. benthamiana* generates high titers of neutralizing antibodies *in vivo*; as such, the protein could be developed as a subunit vaccine against CSFV.

**Electronic supplementary material:**

The online version of this article (10.1007/s10529-020-02892-3) contains supplementary material, which is available to authorized users.

## Introduction

Classical swine fever (CSF), a highly contagious and serious disease of pigs (Edwards et al. 2000), is caused by the CSF virus (CSFV), which is a positive-strand RNA virus belonging to the *Pestivirus* genus within the Flaviviridae family (Moennig 2000). The disease is endemic in Asia, Eastern Europe, and the Americas, as well as in some African countries (Greiser-Wilke et al. 2000; Tu et al. 2001; Deng et al. 2005; Cha et al. 2007; Postel et al. 2013). In Korea, live vaccines have been used traditionally to control swine fever where all pigs must be vaccinated early in life (Lim et al. 2016). Although the vaccine works and is inexpensive, it lacks stability, and it is hard to distinguish between pigs infected with swine fever and pigs that have been vaccinated (the DIVA concept; differentiating **i**nfected from vaccinated animals), which limits the pig trade. Because of this, DIVA vaccines have emerged as important lines of study and many groups have focused on generating marker vaccines.

CSFV has a genome of about 12.3 kb, which encodes a 3898 amino acid polypeptide from which four structural proteins (C, Erns, E1, and E2) and eight nonstructural proteins (Npro, p7, NS2, NS3, NS4A, NS4B, NS5A, and NS5B) are generated (Meyers et al. 1996). Of the four CSFV structural proteins, the E2 glycoprotein is the main target of neutralizing antibodies generated during CSFV infection and is considered a major antigenic protein in the vaccine market (Ahrens et al. 2000; Bouma et al. 1999; Dewulf et al. 2000). CSFV subunit vaccines have been developed using recombinant E2 proteins expressed in insect cells (Ahrens et al. 2000; Bouma et al. 1999, 2000) and, BAYOVAC® CSF Marker (Advasure®, Pfizer, UK) and Porcilis® Pesti (IDEXX CSF marker, IDEXX Europe B.V., the Netherlands) are commercially available in Europe market. Although they were shown to provide protection against CSFV infection, there were reports that they didn’t efficiently provide early protection and transplacental transmission (Depner et al. 2001; van Oirschot 2003). In addition to insect cells, other systems using yeast and mammalian cells are also used to produce recombinant E2 proteins (Lin et al. 2012; Hua et al. 2014). However, large-scale manufacturing of these expression system remain expensive. The genotypes of CSFV can be classified into three major groups with several subgroups (Paton et al. 2000; Postel et al. 2013) and it has reported that antibodies specific to one genotype E2 might not provide protection to other CSFV genotypes (Luo et al. 2013). Therefore, in order to effectively protect against the prevalent CSFV in south Korea, it is necessary to develop a vaccine using the E2 protein for this genotype.

There are many bioreactors that produce pharmaceutical proteins. Among other things, plant-based production of recombinant proteins is an attractive platform because plants are easy to grow, produce large amounts of protein, and do not require expensive facilities (Nandi et al. 2016; Sabalza et al. 2014; Lomonossoff and D’Aoust 2016; Rybicki 2017). The E2 protein produced by *Arabidopsis thaliana* reacts with antibodies against the native E2 protein. In addition, *Arabidopsis thaliana*-derived E2 antibodies in a mouse can recognize the native E2 protein suggesting that structure of plant-produced E2 is comparable to native E2 protein (Sohn et al. 2018). In addition, transient expression of recombinant E2 protein from the leaves of *Nicotiana benthamiana* or from the leaves of transgenic *N. benthamiana* lines generates antibodies that can neutralize CSFV in pigs and protect them against subsequent challenge with CSFV (Laughlin et al. 2019; Park et al. 2019).

Immunoglobin (Ig)G, one of the most abundant proteins in plasma, has a long half-life. The tail (Fc region) of these immunoglobulin molecules modulates immune cell activity by interacting with cell surface receptors and with proteins belonging to the complement system (Nimmerjahn and Ravetch 2008). Fusion of the Fc domain with a specific antigenic protein ensures an immune response to that antigen. The main reason for fusing proteins to the Fc domain is to enhance solubility and the half-life of an antigenic protein (Kontermann 2011; Levin et al. 2015). In addition, the Fc region enables easy and cost-effective purification of recombinant protein by protein A chromatography (Carter 2011; Ghose et al. 2006; Huang 2009).

Here, we asked whether a fusion of the CSFV E2 protein and the Fc domain of porcine IgG could be produced in *N. benthamiana* at low cost. The resulting recombinant protein formed an oligomer rather than a dimer, and injection into mice and pigs generated neutralizing antibodies specific for CSFV.

## Materials and methods

### Plant growth condition

Plants were grown under a 16:8 h light:dark cycle in a growth room maintained at 25 ± 2 °C and 50 ± 5% relative humidity.

### Plasmid construction

We used the pCAMBIA1300 MELCHE2 construct (Park et al. 2019) that contains cellulose binding domain (CBD)-fused E2 as a backbone. The CSFV E2 coding sequence was derived from GenBank (Accession number YP_009508222, amino acid positions 691-1030) and a codon-optimized synthetic gene was purchased (Bioneer Inc. Daejeon, Korea). The pCAMBIA1300 MELCHE2 construct was digested with endonucleases *Xma*I and *Sac*I to remove the TEV:CBD:HDEL domain. The pFc2 coding sequence from GenBank (BAM66310.1) was codon-optimized for expression in *N. benthamiana*. Then, it was amplified by polymerase chain reaction (PCR) using the following primers: *Xma*I/pFc2-F, which contains a *Xma*I restriction site and 21 nt of the pFc2 coding sequence, and the HDEL/pFc2-R reverse primer, which contains 12 nt of ER retention signal (His-Asp-Glu-Leu) and 21 nt of the pFc2 coding sequence. Since the HDEL sequence contains a *Sac*I restriction site, the PCR product was digested with *Xma*I and *Sac*I, and ligated into pCAMBIA1300 MELCH to generate *pCAMBIA1300::pmE2:pFc2:HDEL*.

### Transient expression of chimeric E2 protein

The *pCAMBIA1300::pmE2:pFc2:HDEL* construct was introduced into *A. tumefaciens* strain LBA4404 by electroporation. Separate cultures of Agrobacterium harbouring *pCAMBIA1300::pmE2:pFc2:HDEL* and Agrobacterium harbouring p38, silencing repressor, were grown overnight in YEB liquid medium. Agrobacterium cells were collected by centrifugation at 3500×*g* for 20 min and resuspended in infiltration buffer (10 mM MES, 10 mM MgSO_4_, 100 µM acetosyringone, pH 5.6) to reach OD_600_ of 1.0 and mixed each in a 1:1 ratio (v/v). Leaf tissues of 5 to 7-week-old *N. benthamiana* plants were co-infiltrated with the Agrobacterium-mixture of suspension cells. Plants were returned to the greenhouse and grown for a further 4 DPI (day post infiltration). For expression analysis of the fusion protein, fresh leaf tissues were ground under liquid nitrogen to a fine powder in protein extraction buffer (50 mM Tris-HCl, pH 7.5, 150 mM NaCl, 0.1% [v/v] Triton X-100). Total soluble proteins (TSP) were extracted from the ground tissue samples. After filtering with Miracloth (EMD Millipore Corp., Billerica MA, USA; Cat. No:475855-1R), lysates were clarified by centrifugation (13,000×*g*) for 20 min and Soluble and insoluble fractions were collected.

### Generation of transgenic plants

The *pCAMBIA1300::pmE2:pFc2:HDEL* construct was introduced into *A. tumefaciens* strain LBA4404 by electroporation. For plant tissue culture, *N. benthamiana* leaves were fragmented into 0.5 cm × 0.5 cm pieces, and incubated for 10 min with *A. tumefaciens* transformed with pCMABIA1300::E2:pFc2 in Murashige and Skoog (MS) liquid medium containing 2.0 mg/l a-naphthaleneacetic acid (NAA) and 0.5 mg/l 6-benzylaminopurin (6-BAP). Next, the culture medium was removed, and the leaf fragments were placed upside down (i.e., basal side up) on solid MS medium (which had the same composition as the liquid medium) and incubated for 3 days. After washing with MS liquid medium, the leaves were placed upside down on MS solid medium containing 1.0 mg/l NAA, 0.5 mg/l 6-BAP, 200 mg/l kanamycin, and 250 mg/l cefotaxime, and incubated in the dark for 7–10 days. Next, leaves were exposed to light, leading to growth of shoots and roots. The plants were transferred to soil and expression of pmE2:pFc2 was analyzed by western blot analysis of leaf extracts prepared by homogenizing leaves in extraction buffer, followed by centrifugation to yield a supernatant. After harvesting seeds, T3 generation transgenic plants were selected in the presence of hygromycin at a segregation ratio of 3:1.

### Protein purification and western blot analysis

Briefly, 0.5 kg (fresh weight) leaves from transgenic plants were harvested and homogenized in a blender (32,000 rpm) in the presence of 1 L protein extraction buffer (50 mM sodium phosphate buffer, pH 8.0, 300 mM NaCl, 100 mM glycine, 0.5% Triton X-100). To remove debris, extracts were centrifuged for 40 min at 20,000×*g* and supernatants were filtered through Miracloth. Extracts were incubated for 1 h with 20 mL Protein A Agarose Resin (Amicogen, Jinju, Republic of Korea; Cat. No: 1010200). Next, the extract and resins were loaded onto a column at a flow rate of 20 mL/min and washed three times with 200 mL washing buffer (50 mM sodium phosphate buffer, pH 8.0, 300 mM NaCl). Recombinant E2 proteins were eluted using elution buffer (100 mM sodium citrate, pH 3.0, 300 mM NaCl), followed by addition of 3 M Tris-Cl to obtain a pH of 7.2. Each fraction was collected and subjected to western blot analysis. Briefly, proteins were run on 10% SDS-PAGE gels, transferred to PVDF (Polyvinylidene difluoride) membranes (Merck Milipore Ltd., Tullagreen Carrigtwohill; Cat. No:IPVH00010), and incubated with an antibody specific for HRP-conjugated swine IgG (Bethyl Laboratoris, Montgomery, USA; Cat. No: A100-250P) or CSFV (Median, Chuncheon, Republic of Korea; Cat. No.9011) coupled with anti-mouse IgG HRP-conjugated (Bethyl, Montgomery, USA). Immunoblotting bands were visualized using SUPEX Solution kit (Neutronex, Goryeong, Republic of Korea; Cat. No. NXECL-2011) as a substrate and images were obtained with a Chemiluminescence Imaging system (Vilber, FRANCE). Bands were visualized with Coomassie Brilliant Blue R-250 (Biosolution, Suwon, South Korea; Cat. No: BC006).

For concentration of purified protein, eluted fraction was subjected to centrifuge at 3000×*g* using Ultrafiltration unit (Sartorius, United Kingdom, Cat. No. VS6021).

### Size exclusion chromatography and native-PAGE

Size exclusion chromatography was performed using the ÄKTA Prime chromatography system and a HiLoadTM 16/60 Superdex 200 pg (GE Healthcare, Madison, WI, United States) column. The column was washed and equilibrated with 120 ml buffer (50 mM Tris-Cl, pH 7.2, 300 mM NaCl, 0.5 mM EDTA) prior to loading of pmE2 proteins at a flow rate of 1.5 ml/min. Absorbance at 280 nm was monitored, and fractions were collected and subjected to 8% polyacrylamide gel electrophoresis (PAGE) without sodium dodecyl sulfate (SDS) under non-reducing conditions. Blue-Dextran (2000 kD), Alcohol dehydrogenase (150 kD), Bovine Serum Albumin (66 kD), Carbonic Anhydrase (29 kD), and Cytochrom C (12.4 kD) for Size exclusion chromatography and Thyroglobulin (669 kD), Ferritin (440 kD), Catalase (232 kD), Lactate dehydrogenase (140 kD), and BSA (67 kD) for PAGE were used as protein molecular-weight standards. The gels were stained with Coomassie Brilliant Blue R-250.

### Immunization of mice with pmE2:pFc2 and ELISA

Mice (C57BL/6J, male, 6 weeks old) were used for immunization. All animals were handled in accordance with the guidelines and protocols approved by the Pohang University of Science and Technology (POSTECH) Animal Care and Use Committee. Mice were kept isolated for 1 week prior to the experiments to ensure that they were healthy. For single dose immunization, mice received a subcutaneous injection of 1 µg pmE2:pFc2 fusion protein in Freund’s complete adjuvant (Sigma, Cat. No. F5881) (Yoshikai et al. 1990). Bloods were collected from Retro-Orbital plexus weekly and tested in an ELISA. For double immunizations, mice received 1 µg pmE2:pFc2 fusion protein in Freund’s complete adjuvant, followed 2 weeks later by a second dose in Freund’s incomplete adjuvant (Sigma, Cat. No. F5506). Blood samples were collected prior to the primary injection and then weekly (weeks 3 to 8) after the first injection.

Blood samples were centrifuged for 15 min at 3000×*g* to obtain sera and the resulting sera were further analyzed by a commercially available ELISA kit (A VDPro® CSFV AB ELISA kit; Median, Chuncheon, Republic of Korea, Cat. No. ES-CSF-01) to detect anti-pmE2:pFc2 antibodies. Briefly, the 96-well microplate coated with CSFV E2 antigen and all kit reagents were placed on a bench and allowed to reach room temperature for at least 30 min before use. Next, serum samples (diluted 1:10,000) were added to the plates in duplicate. Positive and negative controls were diluted 20-fold. Samples (100 µL) were added to the plate and incubated at 37 °C for 1 h. Next, 100 µL horseradish peroxidase (HRP)-conjugated anti-mouse IgG (1:5000 dilution) was added to each well for 1 h at 37 °C. After rinsing with wash buffer, 100 µL ABTS substrate was added to each well, followed by incubation for 10 min at room temperature. The reaction was stopped by addition of 100 µL Stop Solution. Absorbance at 405 nm was read in an ELISA Reader (Thermo Fisher Scientific, MULTISKAN FC, Cat. no. N07710). Sample to positive ratio (S/P value) was calculated by (OD _sample_ − OD _negative control mean_)/OD _positive control mean_. Test samples with an S/P ratio ≥ 0.14 are positive; those with an S/P ratio < 0.14 are negative. The experiments were repeated three times and presented a representative result.

### Vaccination of pigs with pmE2:pFc2

All experiments involving pigs complied with the current laws of South Korea. Animal care and treatment were conducted in accordance with guidelines established by the Animal and Plant Quarantine Agency Animal Care and Use Committee (QIA-ACUC). The study was approved by QIA-ACUC (permit number 2017-369).

Six piglets (aged 40 days) were allotted randomly to a control group (n = 2) or a vaccinated group (n = 4). The vaccine was prepared by simple hand mixing of 150 µg pmE2:pFc2 with an oil-in-water emulsion adjuvant (ISA 15A VG; SEPPIC MONTANIDE™, Paris, France). The final volume was 1 ml, which was injected intramuscularly. Piglets from the control group were injected with the same volume of PBS. Pigs received a booster vaccination 20 days after the primary vaccination (the same vaccine formulation was used). Blood samples were collected on Day 0, immediately before the second vaccination, and at 20, 60, 90, and 110 days after the second vaccination; neutralizing antibody responses were then examined.

### Serum anti-CSFV neutralizing antibody assay

Sera were tested for the presence of anti-CSFV neutralizing antibodies using a neutralizing peroxidase linked assay in accordance with the standards set out in the manual of the OIE (OIE 2013). In brief, sera were serially diluted to twofold (1:2 to 1:2048) in serum-free MEM media containing 1 µg/ml trypsin and 50 µl of aliquots were added in a 96-well plate. An equal volume of 200 TCID_50_/mL of CSFV LOM strain was added to each well and incubated for 1 h at 37 °C in 5% CO_2_. 100 µl of CPK (cloned porcine kidney) cells (1 × 10^6^/ml) was added to each well and incubated for 3 days at 37 °C in 5% CO_2_. The cells were fixed with 100 µl of pre-chilled 80% acetone for 7 min at − 20 °C. After drying a plate at 37 °C, commercial anti-LOM antibody (Median Diagnostics; Cat. No. 9011) (100 µl of 200-fold dilution) was added and incubated for 1 h at 37 °C. After rinsing out the plate with PBS three times, biotinylated goat anti-mouse IgG antibody (Vector Lab; Cat. No. BA-9200) (100 µl of 200-fold dilution) was added and incubated for 1 h at 37 °C. After washing three times with PBS, VECTASTAIN ABC-HRP Kit (Vector Lab; Cat. No. PK-4000) was added according to the manufacturer’s instructions and incubated for 1 h at 37 °C. After washing three times with PBS, ImmPACT DAB Peroxidase (HRP) Substrate (Vector Lab; Cat. No. SK-4100) was added according to the manufacturer’s instructions. Neutralizing antibody titers in serum samples were expressed as the reciprocal of the highest dilution that yielded 50% neutralization.

## Results

### Generation of the pmE2:pFc2 fusion construct and protein expression in ***N. benthamiana***

**Fig. 1:**
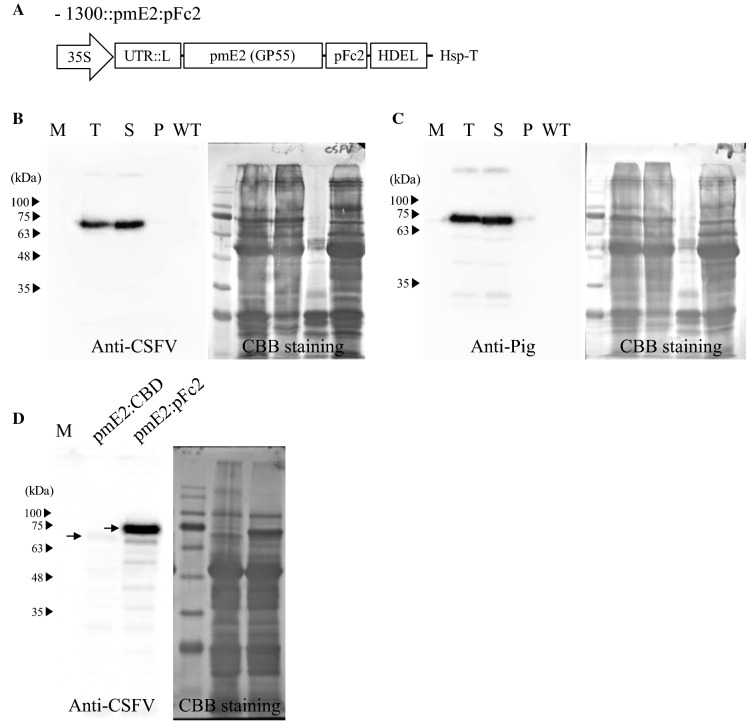
Construction of 1300::pmE2:pFc2:HDEL and expression in *N. benthamiana*. ***a*** Schematic showing the *1300::pmE2:pFc2:HDEL* construct. 35S, double cauliflower mosaic virus 35S promoter; UTR::L, 5′ untranslated region and BiP endoplasmic reticulum (ER)-leader sequence; pmE2, transmembrane domain-deleted classical swine fever virus envelope glycoprotein E2; pFc2, IgG heavy chain constant region from *Sus scrofa*; HDEL, ER retention signal; Hsp-T, *Arabidopsis* HSP terminator. **b** and **c** Western blot analysis of expression and solubility of the recombinant protein. Total protein was prepared from leaves and separated to soluble and insoluble fractions by centrifugation at 20,000×*g* for 15 min. Each fraction was subjected to western blotting with an anti-CSFV antibody and an anti-Pig antibody. The membrane was stained with Coomassie Brilliant Blue. *M* protein markers (molecular weights in kD are shown on the left), *T* plant total extract, *S* soluble fraction, *P* pellet fraction, *WT* wild-type plants. **d** Comparison of expression levels of CBD- or pFc2- fused pmE2. Total extracts were subjected to western blot analysis using anti-CSFV antibody. The arrows indicate recombinant pmE2 proteins

To investigate whether the recombinant pmE2:pFc2 (porcine Fc fragment) fusion was effective as a vaccine, we first constructed a DNA construct for expression in plants. The E2 protein was expressed at high levels after targeting to the endoplasmic reticulum (ER). This was achieved by adding an upstream BiP leader sequence and a downstream HDEL ER retention signal. In addition, we inserted 17 nucleotides into the 5′ UTR region and an *Arabidopsis* HSP terminator into the 3′ UTR region to increase expression (Kim et al. 2014; Nagaya et al. 2010). Previously, we tested three different Fc domains (pFc1, pFc2, and pFc3) from *Sus scrofa* and found out that pFc2 showed strongest expression in *N. benthamiana* (data not shown). Therefore, we fused the pFc2 domain downstream of the pmE2 coding sequence to generate the pmE2:pFc2 fusion construct (Fig. 1a). The pFc2:pmE2 fusion protein generated by transient expression in *N. benthamiana* leaves using *Agrobacterium tumefaciens* was detected in the soluble fraction, indicating that pFc2 increases the solubility of the antigenic protein (Fig. 1b, c). When we looked more closely, we noted a strong band at around 70 kD. The expected size of the E2 recombinant protein is 64 kD; therefore, the strong band appeared to be larger than expected. We assume that this is due to N-glycosylation since E2 protein has putative 7 N-glycosylation sites (Laughlin et al. 2019). Consistent with this, previous studies reported that CBD-fused E2 recombinant proteins have several N-glycosylation sites and are larger than expected when expressed in plants (Park et al. 2019; Sohn et al. 2018). In addition, the pFc2 domain contains one N-glycosylation site suggesting that the larger size is caused by N-glycosylation of both pmE2 and pFc2. To test expressional superiority of the pFc2 fusion, we compared expression levels of pmE2:pFc2 with cellulose-binding domain (CBD):pmE2 by western blotting (Fig. 1d). CBD is derived from *Clostridium thermocellum* and binds to cellulose; therefore, it can be used as a tag for protein purification (Park et al. 2019). As a result, the amount of pmE2:pFc2 was much higher than that of CBD-fused pmE2 when total extracts were prepared from identical fresh weight of leaves. There results suggested that pFc2 fusion increases the expression level of pmE2 protein as well as solubility.

### Generation of pmE2:pFc2 in transgenic ***N. benthamiana***

**Fig. 2:**
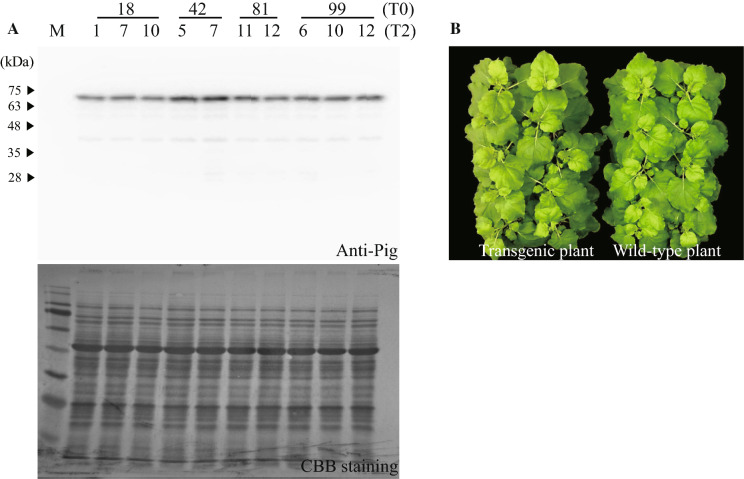
Selection of 1300::pmE2:pFc2:HDEL-expressing transgenic plants. **a** Western blot analysis of 1300::pmE2:pFc2:HDEL expression in transgenic plants (T2 generation). The same amount of total protein extract was prepared from two or three independent lines of four T2 generation transgenic plants and subjected to immunoblotting with an anti-Pig antibody. The blot was visualized by staining with Coomassie Brilliant Blue in order to verify the clarity of total protein extraction processes. M, protein markers; upper numbers indicate transgenic lines at T0; bottom numbers indicate transgenic lines at T2. **b** Phenotypical comparison of wild-type and transgenic plants. Plants were grown side by side from the same germination stage and the morphology was compared at 37 days-of-age

Next, we tried to generate pmE2:pFc2 in transgenic plants to ensure more consistent expression. Similar to transient expression, we performed tissue culture using *A. tumepaciens* after introduction of pCAMBIA 1300::E2:pFc2. We obtained dozens of T0 lines. Western blot analysis revealed that several lines expressed high amounts of recombinant protein; therefore, we selected homogenous lines from T2 plants. To select the most strongly expressed line among four T2 generation plants, we performed further western blot analysis and finally obtained total extracts from two or three lines (Fig. 2a). Expression of recombinant protein was strongest in transgenic line 42. When we compared this transgenic plant with a wild-type plant, we found no significant difference in biomass. Therefore, we used this line to generate recombinant protein (Fig. 2b).

### Purification of recombinant pmE2:pFc2

**Fig. 3:**
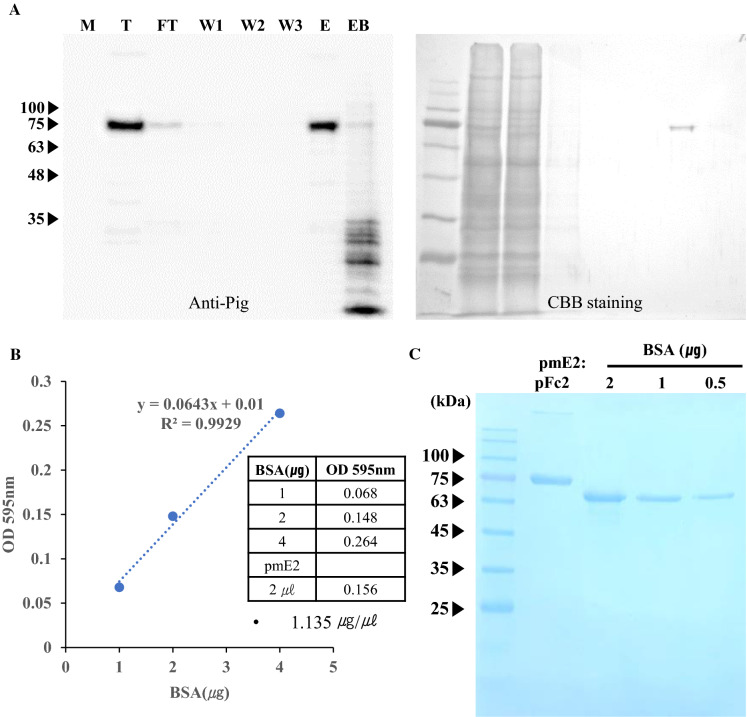
Purification and quantitative analysis of the pmE2 fusion protein. **a** Purification of the pmE2:pFc2 recombinant protein by protein A chromatography. Total extracts were prepared from transgenic plant leaves by incubation in extraction buffer, followed by centrifugation. Samples were loaded onto a column containing protein A resin. After binding, resin-bound recombinant proteins were washed three times and then eluted in elution buffer. Each fraction was separated by SDS-PAGE and subjected to western blot analysis with an antibody specific for anti-pig IgG (whole). *T* total fraction, *FT* flow-through, *W1-3* wash-off fractions, *E* elution fraction, *EB* post-elution resin content. **b** Quantification of purified pmE2:pFc2. Purified protein from **a** was concentrated by centrifugal filtration, and subjected to perform Bradford assay. Linear regression was generated by using 1, 2 and 4 µg of bovine serum albumin (BSA) and 2 µg of pmE2:pFc2 was used for quantification. **c** Quantification of pmE2:pF2 by SDS-PAGE. 2 µg of pmE2:pFc2 and simultaneously, 0.5, 1, and 2 µg bovine serum albumin (BSA) was run for comparison. The gel was visualized by Coomassie Brilliant Blue staining and band intensities were compared

To purify pmE2:pFc2 recombinant protein, total protein extracts were prepared from the leaves of the transgenic plant using protein extraction buffer. The pmE2:pFc2 fusion was purified by affinity chromatography on protein A beads. All fractions including total, flow-through contained unbound E2, wash-off and elution were monitored by western blot analysis (Fig. 3a). Compared with the total protein extract, the flow-through fractions contained around 10% unbound E2:pFc2. In addition, the first-wash fraction exhibited only faint bands, while the second- and third-wash fractions contained very little E2, indicating tight binding of E2:pFc2 to protein A beads. Almost all the E2 protein was present in the eluted fraction; very little remained on the beads after elution, indicating that the elution buffer stripped the beads effectively.

Producing a vaccine from a recombinant protein requires a concentration step to generate a high concentration of antigenic protein in a small volume. Therefore, purified proteins must be concentrated, a process that can result in loss of protein due to aggregation. When we concentrated the eluted fraction by centrifugal filtration, it was enriched without aggregation. Next, the proteins were loaded onto SDS-PAGE gels along with known concentrations of bovine serum albumin (BSA) (Fig. 3b). We estimated that the amount of pmE2 protein was about 1.135 µg/µl, and that 302 mg pmE2 protein could be produced from 1 kg plant leaves.

### Characterization of pmE2:pFc2

**Fig. 4:**
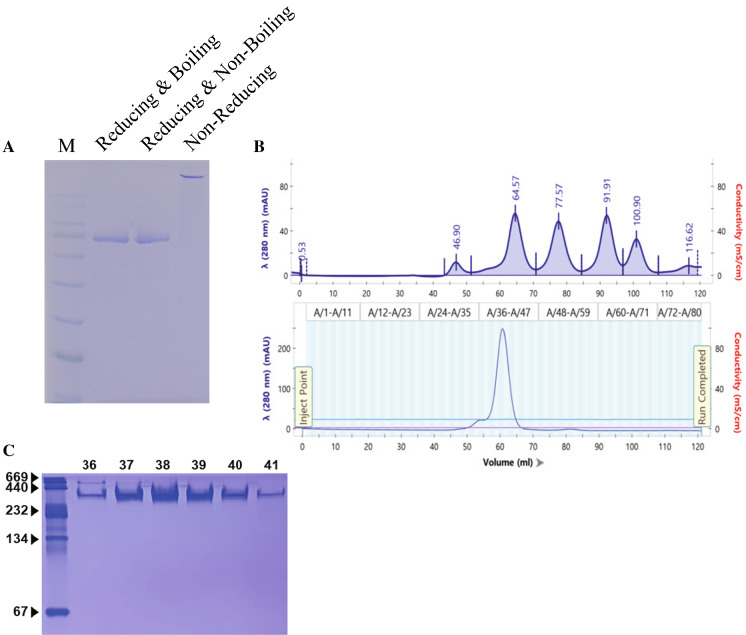
Characterization of pmE2:pFc2 recombinant Status of pmE2:pFc2 under different conditions. Purified protein was treated with β-mercaptoethanol and/or boiling, and then separated by SDS-PAGE. The gel was visualized by Coomassie Brilliant Blue staining. **b** Size exclusion chromatography of pmE2:pFc2. Purified recombinant proteins were injected onto a size exclusion column and separated (bottom panel). Blue-Dextran (2000 kD), Alcohol dehydrogenase (150 kD), Bovine Serum Albumin (66 kD), Carbonic Anhydrase (29 kD), and Cytochrom C (12.4 kD) were used as protein molecular-weight standards (upper panel). **c** Native-PAGE analysis of recombinant proteins eluted from the column. The fraction corresponding to the major peak from **b** was separated on 8% Native-PAGE gels and visualized by CBB staining

Previous reports suggest that the CSFV E2 protein forms a dimer (Lin et al. 2009; Hua et al. 2014; Risatti et al. 2007; Thiel et al. 1991). However, we showed previously that the E2 protein fused to a CBD was expressed in plants as an oligomer (Park et al. 2019). Moreover, pFc2 forms dimers via the tail part of the antibody (Janeway et al. 2001). Thus, we performed SDS-PAGE under reducing and non-reducing conditions to investigate whether purified pmE2 protein forms dimers or oligomers when expressed in plants. We observed a band that was much larger than that expected for the protein only under non-reducing conditions (Fig. 4a). This indicates that the recombinant protein was multimeric. To confirm this, we subjected the purified protein to size exclusion chromatography (Fig. 4b). We observed a small peak at 54 ml and a major peak at 60 ml; no peak was observed in the fraction corresponding to a monomer (78 ml). The major peak was slightly larger than the peak corresponding to 150 kDa (64.18 ml). To confirm that these peaks were pmE2, each fraction from each peak was analyzed by native-PAGE; bands were observed at sizes corresponding to a tetramer or a pentamer (Fig. 4c). By contrast, no protein was in the fraction corresponding to the peak at 54 ml (data not shown). This result is consistent with data derived from CBD-fused pmE2. Therefore, we believe that these characteristics are due to pmE2 itself, rather than to the tags, when expressed in plants.

### Immunogenicity of pmE2:pFc2 in mice

**Fig. 5:**
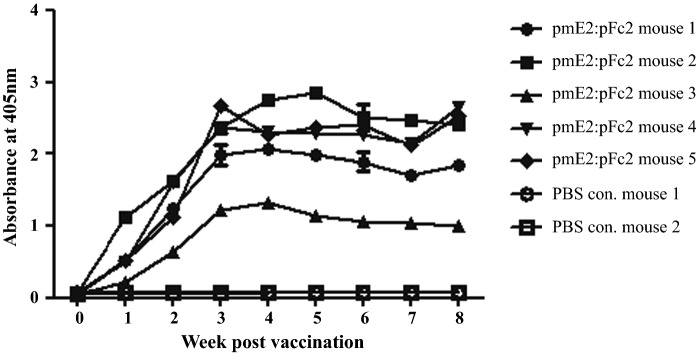
Immunogenicity of pmE2:pFc2 in mice. PmE2:pFc2 fusion protein (1 µg) combined with Freund’s complete adjuvant was injected into mice. Sera were collected every week post-vaccination and subjected to ELISA. The negative control comprised the volume of PBS. Signal intensity was measured at A405

Prior to testing the vaccine in pigs, we used pmE2:pFc2 to immunize mice to assess whether recombinant pmE2 protein produced from *N. benthamiana* acts as a vaccine. Mice (C57BL/6J, male, 6 weeks old) received a subcutaneous injection of 1 µg pmE2:pFc2 in complete Freund’s adjuvant or the same volume of PBS (as a control). After one-dose injection, serum samples were collected on a weekly basis and antibody levels were tested in an ELISA (Fig. 5). Anti-CSFV antibody levels in all mice increased gradually, peaking at 4–5 weeks. These levels were maintained or decreased slightly until week 8 indicating that the chimeric protein produced from plant acts as an antigen. In addition, we used mouse serum (week 8) to test for neutralizing antibodies (Table 1) in order to investigate whether the antibody against recombinant pmE2 can provide protection against CSFV. Most sera had neutralizing antibody titers of 2^7^–2^9^, although slight variations were observed. This level of neutralizing antibodies suggests that the fusion protein has great potential as a vaccine.

**Table 1:** Single- and double-dose vaccination and classical swine fever virus (CSFV) neutralizing antibody titers from mouse serum

Vaccination	Antigen	Mouse ID#	VNA titer
Single-dose vaccination	pmE2:pFc2	1	128
		2	256
		3	32
		4	256
		5	512
Double-dose vaccination	pmE2:pFc2	1	256
		2	256
		3	64
		4	128
		5	64
Back titers			177
Negative control			< 4
Positive control			2048

*VNA* virus neutralizing antibody, *CSFV* neutralizing antibody titers were measured as described in the Materials and methods section

### Verification of CSFV antibody-mediated neutralization in serum from pigs immunized with pFc2:pmE2

Finally, piglets were injected with 150 µg pmE2:pFc2 in ISA 15A VG, and neutralizing antibody responses were verified by testing serum in a virus neutralization test (Table 2). Four of six pigs (aged 40 days) were vaccinated with 150 µg pmE2:pFc2 and then boosted 20 days later with the same vaccine formulation. The other two pigs were injected with the same volume of PBS (control). Serum was obtained from each pig at 20, 60, 90, and 110 days post-second vaccination and neutralizing antibody titers were measured. Although some neutralizing antibodies were detected as early as 20 days after the primary vaccination, a marked increase in titer was observed at 20 days post-booster vaccination. The high neutralizing antibody titers were maintained at values > 6 (log2) for 170 days, although they fell gradually after reaching a peak at aged 80 days. Thus, pmE2:pFc2 generated neutralizing antibodies specific for CSFV in the natural host.

**Table 2:** CSFV-neutralizing antibody responses in pmE2:pFc2 vaccinated piglets

	Age	40-day	60-day	80-day	110-day	150-day	170-day
	Piglet ID#	1st injection	2nd injection	20 days post-boosting	60 days post-boosting	90 days post-boosting	110 days post-boosting
PBS control	1	0	0	0	0	0	0
	2	0	0	0	0	0	0
Ave. VNA titers (log_2_)		0	0	0.0	0.0	0.0	0.0
pmE2:pFc2	35	0	3	10	8	6	6
	36	0	8	11	7	7	6
	38	0	6	10	7	6	5
	39	0	5	12 <	8	8	7
Ave. VNA titers (log_2_)		0	5.5	10.75	7.5	6.75	6

*VNA* virus neutralizing antibody, *CSFV* neutralizing antibody titers were measured as described in the Materials and methods section

## Discussion

Most CSFV vaccines in use today in CSF-endemic areas, including Asia, are modified live vaccines (MLV); although MLVs provide good protection against CSFV infection, it is difficult to distinguish between infected and vaccinated pigs (Blome et al. 2006; Deng et al. 2005; van Oirschot 2003). Therefore, it is difficult to prove that the area is free from this disease, which constrains import and export of pigs. To solve these problems, subunit marker vaccines are being developed. Many studies are examining the efficacy of vaccines based on the CSFV E2 glycoprotein; indeed, some products have come to market: BAYOVAC® CSF Marker (Advasure®, Pfizer, UK) and Porcilis® Pesti (IDEXX CSF marker, IDEXX Europe B.V., the Netherlands). The two viral glycoproteins E2 and E^rns^ are necessary for viral attachment to host cells; of these, E2 is the major envelop glycoprotein that induces production of anti-CSFV neutralizing antibodies (König et al. 1995; Weiland et al. 1990). Previously, we demonstrated that an E2 glycoprotein fused with a CBD was immunogenic in pigs and confirmed that it provided efficient protection against CSFV challenge (Park et al. 2019). Here, we intended to use the recombinant protein Fc domain from pig IgG as an alternative to the CBD and evaluate its efficacy in animals. An ideal vaccine should not only be efficient and safe, but also affordable for end-users. Simple and successful expression of large amounts of recombinant protein with high immunogenicity is the key to cost-effectiveness. This is why we chose the porcine IgG Fc domain as the fusion partner. The Fc domain improved solubility and increased expression of E2 when expressed in plants. In addition, the Fc domain makes isolation of recombinant proteins by protein A affinity chromatography both easy and cheap. Although protein A resin is not cheap, appropriate use and management through a cleaning-in-place protocol means that hundreds of purification cycles can be performed without disassembly of the equipment, resulting in significant economic benefits (Gronberg et al. 2011; Zhang et al. 2017). The most important advantages of the Fc domain are that it increases the half-life of an antigenic peptide in plasma and interacts with Fc receptors on immune cells, thereby acting as a molecular adjuvant. Taking advantage of these benefits, we produced 302 mg recombinant pmE2 protein from 1 kg tobacco leaves. This is around tenfold higher than that reported previously using CBD:pmE2 (30.3 mg/kg) (Park et al. 2019). Although the benefits of low manufacturing costs have been mentioned in producing recombinant proteins from plants, there has been much debate due to the lack of research. There are a few techno-economic analyses for producing recombinant proteins in plants (Buyel anf Fisher 2012; Tuse et al. 2014; Walwyn et al. 2015; Nandi et al. 2016). These simulation reports suggest that cost-effective production with plant expression system can be achieved over alternative platforms although it depends on the products. In addition, high expression of recombinant proteins contributes to lower capital requirements and cost of final products. Therefore, the subunit vaccine based on pFc2-fused pmE2 chimeric protein can help to provide protection against CSFV at a lower price.

Previous reports show that E2 protein exists as a dimer, as does the Fc domain. However, we found that pFc2:pmE2 was expressed as a multimer. Consistent with this, when CBD-fused E2 was expressed in plants, the product was also multimeric (Park et al. 2019; Sohn et al. 2018). According to size-exclusion chromatography, the protein seemed to form trimer considering that the peak was slightly larger than the peak corresponding to 150 kD. However, native-PAGE displayed the band corresponding to tetra or pentamer size. Because the migration of the protein on native-PAGE is affected by diverse factors such as size, folding and isoelectric point, it may not perfectly match with molecular markers. Therefore, more research is needed to establish the structure of pmE2:pFc2, and to examine how it affects the immunogenicity of recombinant proteins and their potential use as vaccines.

In this study, we used pmE2:pFc2 expressing transgenic plants instead of transient expression system. Transient expression generally has been shown to give high amount of target protein than stably expressing transgenic plants (Yamamoto et al. 2018) and we also obtained same results with this fact (data is not shown in here). Thus, assuming that transient expression uses the same biomass, more protein can be produced in a short period and many plant-based pharmaceutical companies are already using the platform. However, there are several reason why we used transgenic plants to produce the recombinant protein. In case of transient expression system, the expression level of target genes is not uniformed. On the other hand, transgenic plant provide stable expression of recombinant genes once the homozygous transgenic lines are generated. This leads to large-scale production easier than transient system. In economic point of view, upstream processes in transient expression system require additional equipment and material for *A.tumefaciens*-mediated infiltration while they can be eliminated in transgenic approach, which enables to produce recombinant proteins more inexpensively (Garabagi et al. 2012). Moreover, transient expression system requires additional step to avoid concerns relating to endotoxins that is caused by the use of *A.tumefaciens* for vacuum infiltration. For these reasons, we think it is more appropriate to use transgenic plants in situations where vaccines must be produced steadily, whereas transient expression system is suitable for urgent situations.

Next, we examined neutralizing antibody responses to CSFV virus in mice and pigs. We found that vaccination of mice and pigs with pmE2:pFc2 generated anti-CSFV neutralizing antibodies. The pig experiments showed a slight increase in the titer of neutralizing antibodies at 20 days post-primary vaccination, and a dramatic increase in titer after the second vaccination. Neutralizing antibody titers were maintained at 6 (log2) until 170 days, although they did fall slightly thereafter. In addition, neutralizing antibodies were readily detectable in mouse serum even at 8 weeks post-vaccination. It is desirable to minimize the number of vaccination to reduce the burden on end users. We confirmed a single vaccination generated antibodies at levels similar to a double vaccination indicating that one-dose injection with 1 µg of pmE2:pFc2 is enough to induce immune responses in mice. Taken together, the results suggest that pmE2:pFc2 induces an efficient neutralized antibody response to CSFV. A limitation is that we vaccinated animals with a single dose (150 µg) and used only one type of adjuvant; further studies should vary both parameters to achieve better results. In addition, further studies should address several important issues. For instance, we need to clarify whether vaccination of pigs affects viral shedding and the viral load. Also, we need to examine effects on vertical and horizontal transmission. In other words, it is important to examine whether vaccinated pregnant sows can prevent transplacental transmission of CSFV to the fetus.

We confirmed the presence of anti-pFc2 antibodies in immunized pigs (data is not shown). Antibodies typically contains complex bi-antennary glycans whereas the heterogenous pFc2 may have high mannose type of glycans due to ER accumulation. N-glycans at CH2 domain influence the folding of the Fc part (Mimura et al. 2001). Therefore, it is possible that pFc2 can be recognized as antigen. Nevertheless, the vaccinated pigs hardly displayed adverse effects during the study. Highly sophisticated approach using glyco-engineering is required for minimizing autoimmune reactions and improved vaccine development based on Fc fragment.

In conclusion, we expressed pmE2:pFc2 in transgenic *N. benthamiana* plants, and isolated and purified the recombinant fusion protein at high yield and low cost. In addition, we confirmed that vaccination of mice and pigs with the fusion protein generated a neutralizing antibody response against CSFV. Taken together, this recombinant protein could be developed as a subunit vaccine against CSFV in a cost-effective manner.

## Electronic supplementary material

Below is the link to the electronic supplementary material.

Supplementary material 1 (PDF 83.1 kb)

## Abbreviations

CSFV: Classical swine fever virus
DIVA: Differentiating infected from vaccinated animals
ER: Endoplasmic reticulum
CBD: Cellulose-binding domain
BSA: Bovine serum albumin
MLV: Modified live vaccines
PCR: Polymerase chain reaction
