# Supplementary material for: A classical swine fever virus E2 fusion protein produced in plants elicits a neutralizing humoral immune response in mice and pigs
Source: Biotechnol Lett. 2020 Apr 22;42(7):1247–61. doi: 10.1007/s10529-020-02892-3 (PMC7223222; doi:10.1007/s10529-020-02892-3)
Supplement: Supplementary file 1 — Supplementary material 1 (PDF 83.1 kb) [file 10529_2020_2892_MOESM1_ESM.pdf]

Supplemental Table 1. The list of nucleotides sequences used in this study

Name	Nucleotides sequence
*Xma*I/pFc2-F	CCCGGGCAGTTGGAAGACCATGCCCTA
HDEL/pFc2-R	GAGCTCATCGTGTTTCCCTTGTGTTTTAGAAAC
